# Exercise-Induced Lactate Release Mediates Mitochondrial Biogenesis in the Hippocampus of Mice *via* Monocarboxylate Transporters

**DOI:** 10.3389/fphys.2021.736905

**Published:** 2021-09-16

**Authors:** Jonghyuk Park, Jimmy Kim, Toshio Mikami

**Affiliations:** ^1^Department of Anatomy and Neurobiology, Graduate School of Medicine, Nippon Medical School, Tokyo, Japan; ^2^Department of Health and Sports Science, Nippon Medical School, Tokyo, Japan

**Keywords:** exercise, hippocampus, peroxisome proliferator-activated receptor gamma-coactivator 1α, mitochondrial biogenesis, lactate, microdialysis

## Abstract

Regular exercise training induces mitochondrial biogenesis in the brain *via* activation of peroxisome proliferator-activated receptor gamma-coactivator 1α (PGC-1α). However, it remains unclear whether a single bout of exercise would increase mitochondrial biogenesis in the brain. Therefore, we first investigated whether mitochondrial biogenesis in the hippocampus is affected by a single bout of exercise in mice. A single bout of high-intensity exercise, but not low- or moderate-intensity, increased hippocampal PGC-1α mRNA and mitochondrial DNA (mtDNA) copy number at 12 and 48h. These results depended on exercise intensity, and blood lactate levels observed immediately after exercise. As lactate induces mitochondrial biogenesis in the brain, we examined the effects of acute lactate administration on blood and hippocampal extracellular lactate concentration by *in vivo* microdialysis. Intraperitoneal (I.P.) lactate injection increased hippocampal extracellular lactate concentration to the same as blood lactate level, promoting PGC-1α mRNA expression in the hippocampus. However, this was suppressed by administering UK5099, a lactate transporter inhibitor, before lactate injection. I.P. UK5099 administration did not affect running performance and blood lactate concentration immediately after exercise but attenuated exercise-induced hippocampal PGC-1α mRNA and mtDNA copy number. In addition, hippocampal monocarboxylate transporters (MCT)1, MCT2, and brain-derived neurotrophic factor (BDNF) mRNA expression, except MCT4, also increased after high-intensity exercise, which was abolished by UK5099 administration. Further, injection of 1,4-dideoxy-1,4-imino-D-arabinitol (glycogen phosphorylase inhibitor) into the hippocampus before high-intensity exercise suppressed glycogen consumption during exercise, but hippocampal lactate, PGC-1α, MCT1, and MCT2 mRNA concentrations were not altered after exercise. These results indicate that the increased blood lactate released from skeletal muscle may induce hippocampal mitochondrial biogenesis and BDNF expression by inducing MCT expression in mice, especially during short-term high-intensity exercise. Thus, a single bout of exercise above the lactate threshold could provide an effective strategy for increasing mitochondrial biogenesis in the hippocampus.

## Introduction

Mitochondrial dysfunction causes neurodegenerative diseases, such as Alzheimer’s disease, and Parkinson’s disease and metabolic diseases, such as type 2 diabetes; it is caused by physiological deterioration owing to aging and lack of exercise (Short et al., 2005; Sutherland et al., 2009; Safdar et al., 2011; Zhang et al., 2012; Rodriguez-Martinez et al., 2013). Mitochondrial biogenesis, the formation of new mitochondria in cells, is vital for mitochondrial function in various tissues. Endurance exercise training induces brain and skeletal muscle mitochondrial biogenesis (Steiner et al., 2011; Halling et al., 2019).

Peroxisome proliferator-activated receptor gamma-coactivator 1-α (PGC-1α) is the master regulator of mitochondrial biogenesis in various cell types (Sutherland et al., 2009; Wareski et al., 2009; Lezi et al., 2013). Eight weeks of treadmill exercise training induces an increase in PGC-1α mRNA expression in skeletal muscle and brain regions, including the cortex and hippocampus, with a concomitant increase in mitochondrial DNA (mtDNA) copy number, enhancing exercise performance (Steiner et al., 2011). Training adaptation reflects the accumulation of the beneficial physiological functions produced from single bouts of exercise; thus, to produce a better exercise training strategy, it is essential to understand the beneficial effects of a single bout of exercise and elucidate the mechanism underlying exercise. However, it remains unclear whether a single bout of exercise would increase mitochondrial biogenesis in the brain.

Research indicates that a single bout of exercise (low, moderate, and high intensity) increases the transcriptional regulators of mitochondrial biogenesis [PGC-1α, mitochondrial transcription factor A (TFAM), and nuclear respiratory factor1 (NRF1) mRNA expression] in mice and human skeletal muscle (Safdar et al., 2009; Little et al., 2010; Tadaishi et al., 2011; Saleem and Hood, 2013; Gidlund et al., 2015), proportional to the exercise intensity used (Ikeda et al., 2008). These results suggest that even a single bout of exercise enhances mitochondrial biogenesis in skeletal muscle, and the extent of its enhancement depends on the intensity of exercise performed. On the other hand, it is unclear whether hippocampal mitochondrial biogenesis is also affected by a single bout of exercise and whether its effect, if any, depends on exercise intensity.

Skeletal muscles release lactate into the blood during exercise; blood lactate concentrations range from ~3mM at rest to ~10mM in mice following high-intensity exercises, which is above the lactate threshold (LT; Hu et al., 2021). LT is the time point when blood lactate concentrations start to rise from the resting level if the subjects continue to exercise with increasing exercise intensity. This time point is thought to express the increased usage of both muscle glycogen and fast muscle fibers (Billat et al., 2005). Lactate was thought to be only a waste product derived from glycolysis metabolism. However, recent studies revealed that circulating blood lactate serves as energy substrates for the skeletal muscle (Takahashi et al., 2020) and enhances skeletal muscle mitochondrial biogenesis (Rowe et al., 2013; Kitaoka et al., 2016). Additionally, lactate crosses the blood–brain barrier (BBB) through monocarboxylate transporters (MCT) in brain cells (Riske et al., 2017; El Hayek et al., 2019) and supplies the energy substrates to neurons (Kobayashi et al., 2019; Lev-Vachnish et al., 2019). For example, 6-week high-intensity interval training (HIIT) increased hippocampal lactate concentration and enhanced mitochondrial biogenesis (Hu et al., 2021). In addition, HIIT also increased hippocampal brain-derived neurotrophic factor (BDNF) expression and protein, which is a necessary regulator for enhancing mitochondrial quality control, and maintaining neuronal function and survival (Freitas et al., 2018; Hu et al., 2021; Okamoto et al., 2021).

Exogenous intraperitoneally (i.p.) or orally administered lactate works the same way as endogenous lactate released from skeletal muscle during exercise. Lactate administrated chronically to mice functioned as a signal molecule that promoted adult hippocampal neurogenesis (Lev-Vachnish et al., 2019). Acute and chronic peripheral lactate administration increased extracellular lactate concentrations in hippocampal tissue, which produced antidepressant-like effects in mice (Carrard et al., 2018). Lactate administered to hippocampal cells *in vitro* enhanced ATP levels, PGC-1α, BDNF protein level, mtDNA copy number, and potentiated mitochondrial function (Hu et al., 2021). We hypothesized that blood lactate increased by high-intensity exercise could promote PGC-1α mRNA expression in the hippocampus and trigger hippocampal mitochondrial biogenesis.

Lactate derived from brain glycogen is critical for neurons. In brief, lactate generated by glycogen degradation in astrocytes is transferred to the neurons *via* MCTs and acts as energy sources for neurons, helping maintain their function; this system is known as the astrocyte-neuronal lactate shuttle (ANLS; Brooks, 2002). The ANLS has been referred to play a key role in lactate transport and neuronal activity (Pellerin et al., 1998), which is involved in the hippocampal function, such as long-term potentiation and long-term memory as well as endurance exercise capacity (Newman et al., 2011; Suzuki et al., 2011; Lev-Vachnish et al., 2019). Matsui et al. found that a bout of acute moderate-intensity exercise increased hippocampal lactate and ATP levels and decreased brain glycogen concentrations, which was abolished by 1,4-dideoxy-1,4-imino-D-arabinitol (DAB) administration, a glycogenolysis inhibitor, and diminished endurance exercise capacity (Matsui et al., 2017). We thus hypothesized that glycogenolysis inhibition during exercise might also attenuate exercise-induced PGC-1α and MCTs mRNA expression in the hippocampus. Furthermore, intracerebral DAB administration decreased neuron lactate supply from astrocytes and was deleterious to rat’s cognitive function (Suzuki et al., 2011; Matsui et al., 2017). These results suggest that lactate derived from brain glycogenolysis is vital for maintaining brain functions. However, no reports exist on whether lactate derived from brain glycogenolysis contributes to mitochondrial biogenesis after high-intensity exercise.

This study assessed the effects of a single bout of low-, moderate-, or high-intensity treadmill exercise on PGC-1α mRNA expression and mtDNA copy number in the mouse hippocampus. Next, we examined whether exogenous lactate administration increased hippocampal extracellular lactate concentration and mimicked the effects of high-intensity exercise. Then, we examined if exogenous lactate or UK5099, a lactate transporter inhibitor, administered before high-intensity exercise alters mouse hippocampus PGC-1α mRNA expression and mtDNA copy number as well as BDNF expression. Finally, we examined whether pre-exercise intra-hippocampal injection of DAB affected the mouse hippocampal PGC-1α and MCTs mRNA expression following high-intensity exercise.

## Materials and Methods

### Ethical Approval

Animal use and procedures followed the National Institute of Health guideline and were approved by the Animal Care and Use Committee of Nippon Medical School (approval no. 30-030). Furthermore, we exerted all efforts to minimize animal pain and discomfort.

### Animals

Eight-week-old male ICR mice (weight: 36–38g; Sankyo Lab, Tokyo, Japan) were used in this study. All mice were housed (5 per cage) in standard transparent mouse cages (29×18×13cm) and provided *ad libitum* access to standard chow (MF; Oriental Yeast Co, Ltd., Tokyo, Japan) and water. Mice selected for surgery were individually housed in the same cages. Room temperature was maintained at 22–24°C with 50% humidity under a 12h light/dark cycle (lights on: 08:00–20:00). Following each experiment, all mice were killed by decapitation using a guillotine without anesthesia, which was the most humane method, taking both animal welfare and data quality into consideration (Pierozan et al., 2017).

### Treadmill Exercise Protocol

**Figure 1:**
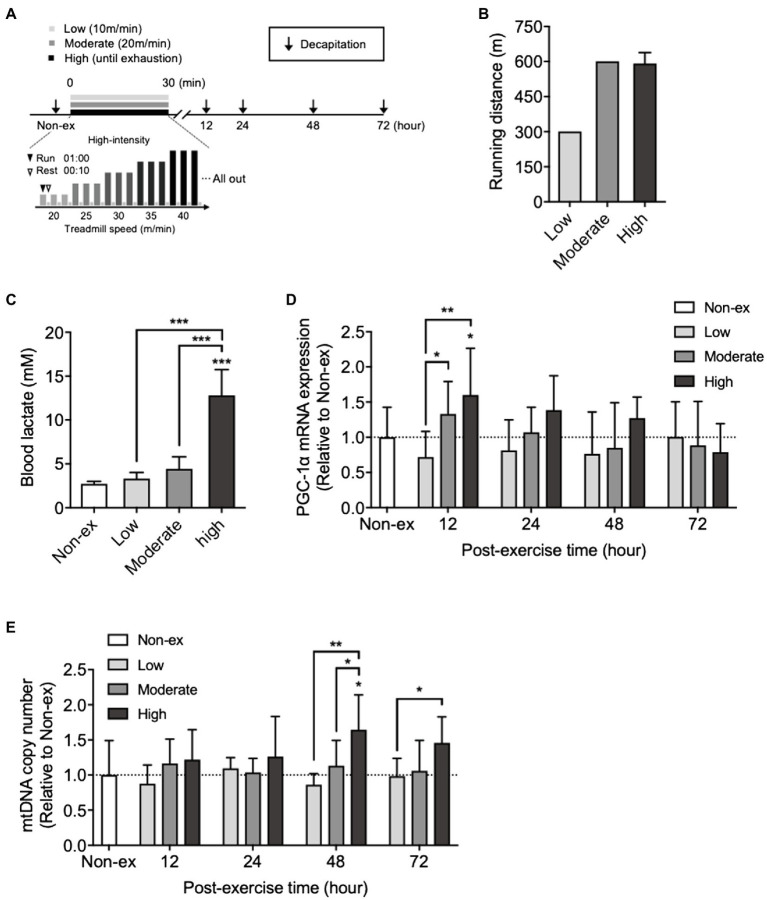
Effects of a single bout of exercise performed at three different intensities on peroxisome proliferator-activated receptor gamma-coactivator 1α (PGC-1α) mRNA expression and mitochondrial DNA (mtDNA) copy number in the hippocampus and on blood lactate levels. **(A)** Protocols for a single bout of exercise. The mice ran on a treadmill at three different intensities: low intensity (low): 10m/min for 30min; moderate intensity: 20m/min for 30min (moderate); and high intensity: the treadmill speed was initially set at 20m/min and gradually increased by 5m/min until the mice were exhausted. **(B)** Running distance for 30min at low- and moderate-intensity exercise groups and exhaustion at high-intensity exercise group. **(C)** Blood lactate levels immediately after a single bout of exercise at three different exercise intensities. **(D)** Time course changes in PGC-1α mRNA expression, and **(E)** mtDNA copy number in the hippocampus at non-exercise (Non-ex), 12, 24, 48, and 72h after three different exercise intensities (Non-ex, *n*=9; low, *n*=8–10/group; moderate, 8–10/group; high, 8–10/group). All data were presented as the mean±SD values. Data were analyzed using one-way ANOVA with Tukey’s *post-hoc* tests or two-way ANOVA with Bonferroni’s *post-hoc* tests. ^*^p<0.05; ^**^p<0.01; and ^***^p<0.001 in comparison with the non-exercise group if not otherwise indicated.

All mice were subjected to 15min of treadmill running at a treadmill running speed of 10–15m/min for three consecutive days during the habituation period. After 3days of rest following the treadmill habituation period, mice performed treadmill running at low, moderate, or high intensity. The protocol for a single bout of exercise at three intensities is shown in Figure 1A. In brief, low- and moderate-intensity exercise group mice were subjected to a single bout of running on a treadmill at speeds of 10 or 20m/min, respectively, for 30min.

High-intensity exercise consisted of intense intermittent running, including rest, on a treadmill with a gradual increase in speed determined according to the method of Lee et al. (2018) as follows: The mice ran for 1min on a treadmill set at a treadmill speed of 20m/min and then rested for 10s, which was defined as one set. After the mice performed three sets at the same treadmill speed, the speed was increased to 25m/min, and the mice again performed three sets. Thus, the treadmill speed continued to rise by 5m/min until exhaustion. Exhaustion was defined as the point at which mice stayed on the grid at the back of the treadmill for a period of 30s despite being given mild touches.

### Microdialysis

A stainless steel guide cannula (EICOM CORP, Japan) was implanted stereotaxically into mice dorsal hippocampus (−1.8mM anteroposterior, ±1.8mM mediolateral, −1.9mM dorsoventral from the bregma) following a previously reported method (Ishikawa et al., 2016). The mice were allowed to recover for 5days after surgery. After that, the microdialysis probe (EICOM, CX-4-01) was inserted into the guide cannula approximately 120min before the experiment. The probe was perfused with Ringer’s solution, containing: 147mM NaCl, 4mM KCl, 2.3mM CaCl2; pH 6.5.

The microdialysis probe was connected to a commercially available microdialysis liquid-swivel (EICOM) to ensure the free movement of the mice. For measuring hippocampal extracellular lactate concentration, ICR male mice (*n*=4) were injected with sodium lactate dissolved in PBS (pH 7.4) at a dose of 2g/kg BW by I.P. injection as previously described (Lezi et al., 2013). Another series of ICR male (*n*=5) mice received the UK5099 (0.1ml; 50μmol/kg BW; Tocris, United Kingdom) 30min before lactate injection. The flow rate was 1μl/min allowing the collection of 10μl samples every 10min. Microdialysis samples were collected into ice-cooled polyethylene tubes (EICOM) using an EFC-96 fraction collector (EICOM). All other microdialysis samples were frozen immediately and stored at −80°C until lactate analysis.

### Time Course of Changes in Blood Lactate Concentration After I.P. Injection

ICR male mice (*n*=6) were injected with sodium lactate dissolved in PBS (pH 7.4) at a dose of 2g/kg BW by I.P. injection. Lactate levels in blood obtained from the tail vein were measured using a portable blood lactate analyzer (Lactate Pro 2, Arkray, Tokyo, Japan) at pre-injection (Pre), 0, 5, 10, 15, 30, 60, and 180min after sodium lactate injection.

### Experimental Design

#### Experiment 1

The effects of a single bout of exercise at low, moderate, or high intensity on blood lactate, hippocampal PGC-1α mRNA, and mtDNA levels are summarized in Figure 1.

ICR male mice were divided into four groups: non-exercise (Non-ex, *n*=9), low-intensity exercise (Low, *n*=8–10/group), moderate-intensity exercise (Moderate, *n*=8–10/group), and high-intensity exercise (High, *n*=8–10/group). Mice blood lactate levels were measured before and immediately after every single bout of exercise. Mice were killed 12, 24, 48, and 72h after each bout of exercise, and the hippocampus was quickly excised, snap-frozen in liquid nitrogen, and stored at −80°C until analysis.

#### Experiment 2

**Figure 2:**
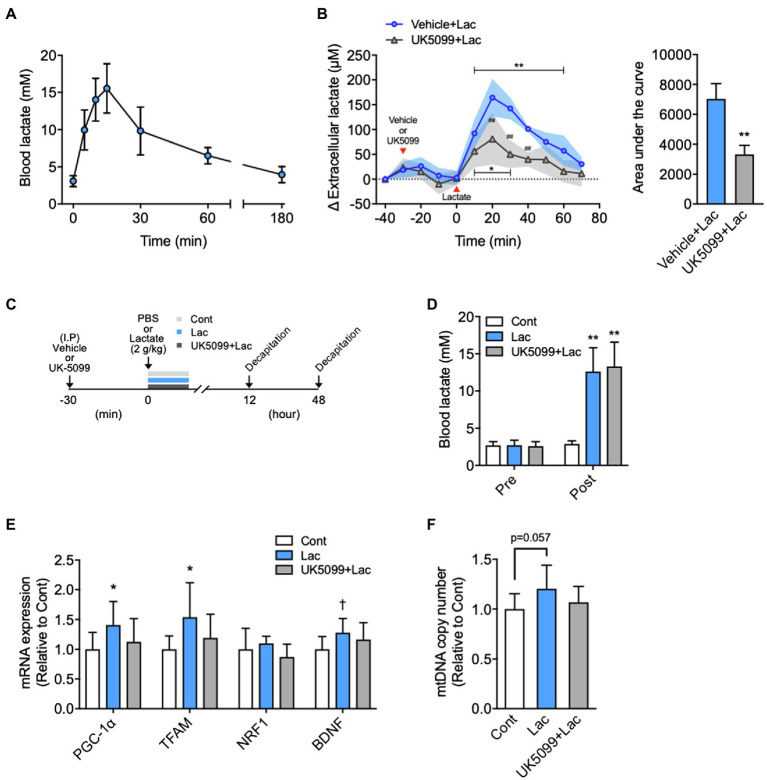
Effects of lactate administration on hippocampal extracellular lactate concentration and mitochondrial biogenesis in the hippocampus. **(A)** Time course changes in blood lactate concentration after I.P. lactate injection (2g/kg; *n*=6). **(B)** The hippocampal extracellular lactate concentration changes after I.P. lactate injection with saline or monocarboxylate transporters (MCT) inhibitor (UK5099) administration and area under the curve (Vehicle + Lac, *n*=4; UK5099+Lac, *n*=5). **(C)** Experimental design: Mice were injected saline or UK5099 by I.P. injection 30min before lactate administration. Blood lactate concentration was measured 15min after I.P. lactate or saline injection. **(D)** Blood lactate concentrations before I.P. lactate injection (Pre) and after lactate injection with and without UK5099 (Post). **(E)** PGC-1α, mitochondrial transcription factor A (TFAM), nuclear respiratory factor1 (NRF1), and brain-derived neurotrophic factor (BDNF) mRNA expression in the hippocampus 12h after I.P. lactate injection with and without UK5099 (Cont, *n*=9; Lac, *n*=9; UK5099+Lac, *n*=8). **(F)** mtDNA copy number in the hippocampus 48h after I.P. injection of lactate with and without UK5099 (Cont, *n*=10; Lac, *n*=10; UK5099+Lac, *n*=8). All data were presented as the mean±SD values. Data were analyzed using two-way ANOVA with Bonferroni’s *post-hoc* tests and unpaired *t*-test (AUC) **(B)** and one-way ANOVA with Tukey’s *post-hoc* tests **(D–F)**. ^*^p<0.05; ^**^p<0.01; and ^†^*p*<0.1 in comparison with baseline or Cont group and ^##^*p*<0.01 compared to Vehicle + Lac group.

**Figure 3:**
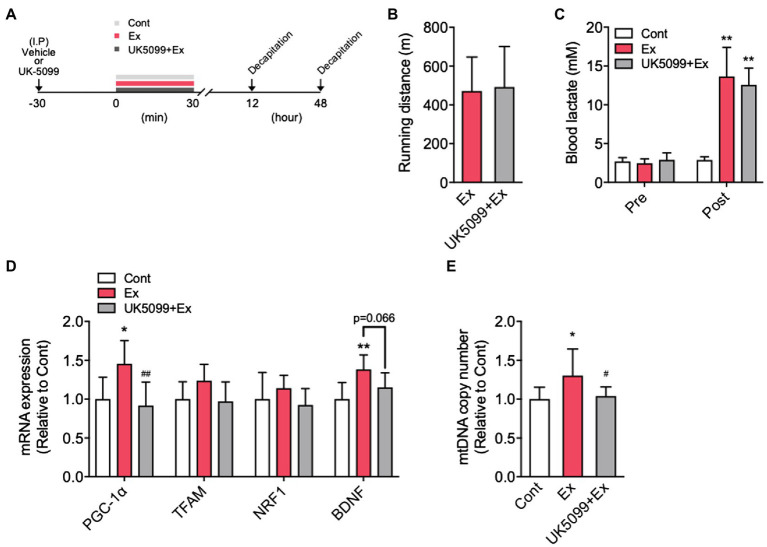
Exercise-induced lactate release increased PGC-1α mRNA expression and mtDNA copy number in the hippocampus. **(A)** Experimental design: Mice were injected with saline or UK5099 by I.P. injection 30min before a single bout of high-intensity exercise. Blood lactate concentrations were measured immediately after exercise. **(B)** Running distance to fatigue on a treadmill for mice administered saline (Ex) or UK5099 (UK5099+Ex) before exercise. **(C)** Blood lactate concentrations before exercise (Pre) and immediately after exercise (Post). **(D)** PGC-1α, TFAM, NRF1, and BDNF mRNA expression in the hippocampus 12h after exercise (Cont, *n*=9; Ex, *n*=10; UK5099+Ex, *n*=8). **(E)** mtDNA copy number in the hippocampus 48h after exercise (Cont, *n*=10; Ex, *n*=10; UK5099+Ex, *n*=10). All data were presented as the mean±SD values. Data were analyzed using one-way ANOVA with Tukey’s *post-hoc* tests. ^*^p<0.05 and ^**^p<0.01 in comparison with Cont group and ^##^*p* < 0.05 and ^##^*p*<0.01 in comparison with Ex group.

**Figure 4:**
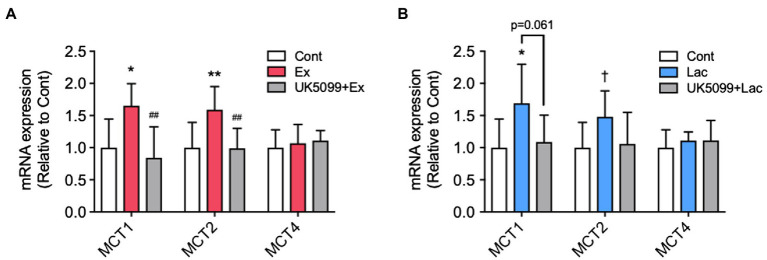
The effects of UK5099 on the hippocampal MCTs and BDNF mRNA expression after lactate administration and high-intensity exercise. Mice were injected with saline or UK5099 by I.P. injection 30min before a single bout of high-intensity exercise and lactate administration. MCT1, MCT2, and MCT4 mRNA expression in the hippocampus 12h after **(A)** exercise (Cont, *n*=10; Ex, *n*=9; UK5099+Ex, *n*=9) and **(B)** I.P. injection of lactate (Cont, *n*=9; Lac, *n*=9; UK5099+Lac, *n*=8). All data were presented as the mean±SD values. Data were analyzed using one-way ANOVA with Tukey’s *post-hoc* tests. ^*^p<0.05; ^**^p<0.01; and ^†^*p*<0.1 in comparison with Cont group and ^##^*p*<0.01 in comparison with Ex group.

The effects of UK5099 administration on blood lactate, hippocampal mitochondrial biogenesis, MCTs, and BDNF mRNA levels after lactate administration or exercise are summarized in Figures 2–4.

ICR male mice were divided into three groups: control (Cont), vehicle + lactate (Lac), and UK5099+lactate (UK5099+Lac). UK5099 (0.1ml; 50μmol/kg BW; Tocris, United Kingdom), or a similar amount of DMSO, was I.P. injected 30min before lactate injection (2g/kg). Another series of ICR male mice were divided into three groups: Cont, vehicle + a single bout of high-intensity exercise (Ex), and UK5099+a single bout of high-intensity exercise (UK5099+Ex). Lactate levels in blood were measured before lactate administration and exercise and then 15min after lactate administration or immediately after exercise. Mice were killed for the measurement of PGC-1α mRNA expression 12h after lactate administration (Cont, *n*=9; Lac, *n*=9; UK5099+Lac, *n*=8) and exercise (Cont, *n*=9; Ex, *n*=10; UK5099+Ex, *n*=8) and for the mtDNA copy number 48h after that administration (Cont, *n*=10; Lac, *n*=10; UK5099+Lac, *n*=8) and exercise (Cont, *n*=10; Ex, *n*=10; UK5099+Ex, *n*=10). The hippocampus was quickly excised, snap-frozen in liquid nitrogen, and stored at −80°C until analysis.

### Surgery for Intra-Hippocampus Injection

After the treadmill habituation period, the mice were anesthetized with isoflurane and positioned in a stereotaxic apparatus for steel cannula placement (Narishige Co., Japan). For intra-hippocampal injection, a small hole was made using a dental drill, and a steel cannula (22-gauge) was inserted into the dorsal hippocampus. The guide cannula was located in the hippocampus (−1.8mM anteroposterior, ±1.8mM mediolateral, and −1.9mM dorsoventral from the bregma) following a previously reported method (Ishikawa et al., 2016) and was fixed to the skull using anchor screws (EICOM) and dental cement. After surgery, the mice were individually housed in a warm cage and allowed to recover completely for at least 3days.

#### Experiment 3

**Figure 5:**
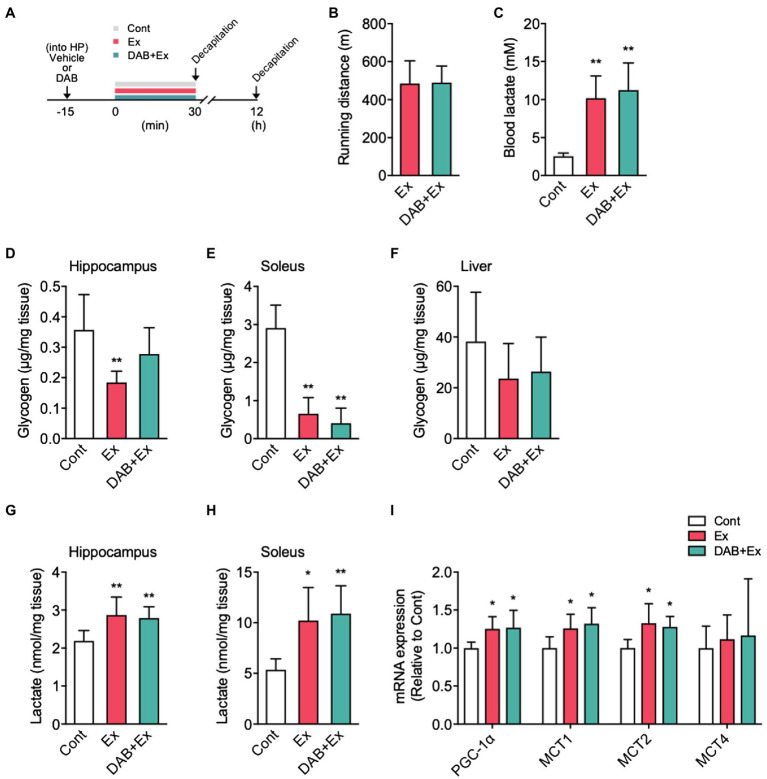
The effects of intra-hippocampal 1,4-dideoxy-1,4-imino-D-arabinitol (DAB) injection before high-intensity exercise on hippocampal glycogen, lactate levels, and PGC-1α, MCTs mRNA expression. **(A)** Experimental design: Mice were injected saline or DAB by intra-hippocampal injection 15min before a single bout high-intensity exercise by using a micro-injection pump. **(B)** Running distance to fatigue on a treadmill for mice administered vehicle (Ex) or DAB (DAB + Ex) before exercise. **(C)** Blood lactate concentrations immediately after exercise and control. **(D)** Glycogen levels in the hippocampus, **(E)** soleus muscle **(F)** liver, **(G)** lactate levels in the hippocampus, and **(H)** soleus muscle immediately after exercise, with vehicle (Ex) or DAB (DAB + Ex) and control (Cont, *n*=6; Ex, *n*=6; DAB + Ex, *n*=6). **(I)** PGC-1α, MCTs mRNA expression in the hippocampus 12h after a single bout of high-intensity exercise (Cont, *n*=6; Ex, *n*=7; DAB + Ex, *n*=7). All data were presented as the mean±SD values. Data were analyzed using one-way ANOVA with Tukey’s *post-hoc* tests. ^*^p<0.05 and ^**^p<0.01 compared to Cont group.

The effects of pre-exercise intra-hippocampal injection of DAB on hippocampal glycogen and lactate concentrations and hippocampal PGC-1α and MCTs mRNA expression are summarized in Figure 5.

After surgery for intra-hippocampal injection, ICR male mice were divided into three groups: control (Cont, *n*=6), saline + a single bout of high-intensity exercise group (Ex, *n*=6), and DAB + a single bout of high-intensity exercise group (DAB + Ex, *n*=6).

DAB (0.25M in 1μl of 0.9% saline; Wako, Osaka, Japan) or 1μl of saline was injected into the hippocampus using a syringe pump (Legato 101, KD Scientific). Fifteen minutes after the injection, mice were subjected to a single bout of high-intensity exercise. Mice were killed by decapitation, and the hippocampus, soleus, and liver were excised immediately after exercise, snap-frozen in liquid nitrogen, and stored at −80°C until analysis. Another series of ICR male mice were divided into Cont (*n*=6), Ex (*n*=6), and DAB + Ex (*n*=6). After intra-hippocampal injection of saline or DAB, mice were subjected to exercise. Mice were killed by decapitation, and the hippocampus was collected 12h after high-intensity exercise, snap-frozen in liquid nitrogen, and stored at −80°C until analysis.

### RNA Extraction and Quantitative Real-Time Polymerase Chain Reaction

The hippocampus samples of mice were homogenized in TRIzol Reagent (Invitrogen, CA, United States) on ice, and total RNA was extracted according to the manufacturer’s instructions. Total RNA was quantified using absorption at 260nm and the 260:280nm ratio to assess concentration and purity. Complementary DNA was synthesized using 1μg of total RNA in a 20μl reaction with the ReverTra Ace^™^ qPCR RT Master Mix with gDNA Remover (FSQ-301; Toyobo, Osaka, Japan) according to the manufacturer’s instructions.

Quantitative real-time PCR was performed with the SsoAdvanced Universal SYBR Green Supermix (Bio-Rad) and a CFX Connect Real-time PCR system (Bio-Rad) to quantify the mRNA levels. Glyceraldehyde-3-phosphate dehydrogenase (GAPDH) was used as the endogenous control. The mouse-specific primers used were as follows: PGC-1α: forward 5′-ACCCTGCCATTGTTAAGACC-3′, reverse 5′-CTGCTGCTGTTCCTGTTTTC-3′; TFAM: forward 5′-GAAGGGAATGGGAAAGGTAGA-3′, reverse 5′-AACAGGACATGGAAAGCAGAT-3′; NRF1: forward 5′- ATCCGAAAGAGACAGCAGACA-3′, reverse 5′- TGGAGGGTGAGATGCAGAGTA-3′; MCT1: forward 5′-TTGTCTGTCTGGTTGCGGCTTGATCG-3′, reverse 5′-GCCCAAGACCTCCAATAACACCAATGC-3′; MCT2: forward 5′-CACCACCTCCAGTCAGATCG-3′, reverse 5′-CTCCCACTATCACCACAGGC-3′; MCT4: forward 5′-TCAATCATGGTGCTGGGACT-3′, reverse 5′-TGTCAGGTCAGTGAAGCCAT-3′; BDNF: forward 5′-TGCAGGGGCATAGACAAAAGG-3′, reverse 5′-CTTATGAATCGCCAGCCAATTCTC-3′; and GAPDH forward 5′-CATCACTGCCACCCAGAAGA-3′, reverse 5′-ATGTTCTGGGCAGCC-3′. The 2^−ΔΔC^_t_ method was used to analyze relative mRNA expression values (Livak and Schmittgen, 2001). Sample analysis for each gene was performed in duplicate.

### mtDNA Copy Number

For quantifying hippocampal mtDNA, total DNA was extracted from the hippocampus using the phenol-chloroform method, as described previously (Mikami et al., 2021). First, the hippocampal tissue (~20mg) was dissolved in 200μl of lysate buffer containing 10mM Tris–HCl (pH 8.0), 150mM NaCl, 10mM EDTA (pH 8.0), 0.1% SDS, and 2.5% proteinase K; next, it was vortexed, incubated at 56°C for 90min, and centrifuged at 2,000*g* for 10min.

The supernatant was mixed with an equal volume of phenol/chloroform/isoamyl alcohol, vortexed, and centrifuged at 2,000×*g* for 10min. Then, the supernatant obtained was mixed with two volumes of 100% ethanol by slow inversion and centrifuged at 10,000×*g* for 10min. After the supernatant was removed, the precipitate was dissolved in 70% ethanol by slow inversion and centrifuged at 10,000×*g* for 10min.

Subsequently, after the supernatant obtained was removed, the precipitate was left for 5min at room temperature, dissolved in Tris-EDTA (TE) buffer (pH 8.0), and stored at −20°C until analysis. DNA concentration and purity were analyzed using absorption at 260nm and the 260:280nm absorption ratio, respectively.

Quantitative real-time PCR (10ng DNA) was performed with the SsoAdvanced Universal SYBR Green Supermix. 18S ribosomal RNA (18S rRNA) was used as the nuclear DNA (nDNA) control. The mtDNA and nDNA primers used were as follows: COX I: forward 5′-TGATTCCCATTATTTTCAGGCTTC-3′, reverse 5′-ACTCCTACGAATATGATGGCGAA-3′; and 18S rRNA: forward 5′-CGCCGCTAGAGGTGAAATTC-3′, reverse 5′-CTTGGCAAATGCTTTCGCTC-3′. The 2^−ΔΔC^_t_ method was used to analyze the relative mtDNA to nDNA copy number ratio (Livak and Schmittgen, 2001). Sample analysis for each gene was performed in duplicate.

### Lactate and Glycogen Concentrations in Tissues

For measuring lactate and glycogen concentrations, the mice were killed immediately after a single bout of high-intensity exercise, and the hippocampus, muscle, and liver tissues were obtained. Glycogen and lactate levels in these tissues were colorimetrically measured using Glycogen Assay Kit II (ab169558; Abcam, Cambridge, United Kingdom) and L-Lactate Assay Kit (MAK064; Sigma, United Kingdom), respectively, according to the manufacturer’s protocols. Samples were deproteinized with a 10kDa MWCO spin filter to remove lactate dehydrogenase.

### Statistical Analysis

Data are expressed as mean±SD and were analyzed using GraphPad Prism version 8.4.1 (MDF Co, Ltd., Tokyo, Japan). Group comparisons were performed using one-way ANOVA with Tukey’s *post-hoc* tests or two-way ANOVA with Dunnett’s and Bonferroni’s *post-hoc* tests. In addition, comparisons of two groups were performed using an unpaired Student’s *t*-test. The differences between groups were considered statistically significant at *p*<0.05.

## Results

### Time Course of Changes in Hippocampal PGC-1α mRNA Levels and mtDNA Copy Number Following a Single Bout of Exercise

We examined the time course changes in hippocampal PGC-1α mRNA and mtDNA copy number after a single bout of exercise at three different intensities (Figure 1A). Low- and moderate-intensity exercise groups ran 300- and 600-meter distances in 30min, respectively. Although running distance until exhaustion in the high-intensity exercise group (time to exhaustion: 26.1±0.37min, including rest) did not differ from the distance covered by the moderate-intensity exercise group (Figure 1B), the blood lactate concentrations immediately after high-intensity exercise were significantly higher than those in the other two groups (*F*_3,36_=80.35, *p*<0.0001; Figure 1C). Hippocampal PGC-1α mRNA levels did not significantly change at any time point after the low-intensity exercise. At 12h after moderate- and high-intensity exercises, the expression levels were significantly higher than those of the low-intensity exercise group. Compared with the non-exercise group, PGC-1α mRNA levels significantly increased only in the high-intensity exercise group (*F*_2,127_=5.26, *p*=0.0065; Figure 1D). The mtDNA copy number significantly increased 48h after a single bout of high-intensity exercise compared with the non-exercise, low-, and moderate-intensity exercise groups (*F*_2,129_=10.70, *p*<0.0001). However, the low- or moderate-intensity exercise groups did not show any increase compared to the non-exercise group (Figure 1E). These results indicated a relationship between the increase in blood lactate and hippocampal PGC-1α mRNA levels observed after high-intensity exercise.

### The Effects of a Single Administration of Lactate on Hippocampal Extracellular Lactate Concentrations and Mitochondrial Biogenesis

Blood lactate levels reached 15.5±1.36mM at 15min following I.P. lactate injection, comparable to that observed immediately after high-intensity exercise (Figure 2A), and returned to baseline levels at 180min after injection. Baseline hippocampal extracellular lactate concentrations did not differ between the two groups (Vehicle + Lac, 99.1±3.71; UK5099+Lac, 107.3±2.44μm; *p*=0.11). The extracellular lactate concentration in the hippocampus was significantly increased following I.P. lactate administration after 10min and remained high until 60min, and then returned to baseline.

At 20min after lactate injection, hippocampal extracellular lactate concentration increased by ~160μm from baseline. Lactate-derived elevation in extracellular lactate concentration was significantly suppressed by UK5099 injection (*F*_1,82_=44.00, *p*<0.0001; Figure 2B). The decrease in hippocampal extracellular lactate *via* UK5099 administration was also proved by the results of the area under the curve analysis (Figure 2B). We administered vehicle or UK5099 to the mice 30min before lactate injection (Figure 2C). We observed no difference in the increased blood lactate levels between Lac and Lac + UK5099 group, which were equal to those observed immediately after high-intensity exercise (Figure 2D). The mice injected with lactate showed significantly higher PGC-1α (*F*_2,23_=3.33, *p*=0.051) and TFAM (*F*_2,23_=3.27, *p*=0.058) mRNA, but not significantly higher in BDNF mRNA (*F*_2,23_=2.61, *p*=0.098). Besides, these mice tended to have a higher mtDNA copy number (*F*_2,25_=2.98, *p*=0.068) in the hippocampus than the Cont group after injection. However, these changes were not observed in mice injected UK5099 (Figures 2E,F).

### The Effects of UK5099 Administration Before Exercise on Hippocampal Mitochondrial Biogenesis

Mice were injected vehicle or UK5099 before a single bout of high-intensity exercise (Figure 3A). Ex and UK5099+Ex group showed a similar running distance to fatigue (Figure 3B) and blood lactate levels immediately after exercise (Figure 3C). However, the Ex group showed significantly higher hippocampal PGC-1α (*F*_2,24_=9.67, *p*<0.001) and BDNF mRNA (12h after exercise; *F*_2,24_=7.89, *p*<0.01), and hippocampal mtDNA copy number (48h after exercise; *F*_2,27_=5.24, *p*<0.05) than the Cont group (Figures 3D,E). In contrast, the UK5099+Ex group showed significantly lower PGC-1α mRNA and mtDNA levels than the Ex group (Figures 3D,E). However, there was no significant difference in downstream regulators of mitochondrial biogenesis, TFAM, and NRF1 mRNA among the groups (Figure 3D).

### The Effects of UK5099 on Hippocampal MCTs mRNA Expression After Lactate Administration and Exercise

We found that MCT1 (*F*_2,24_=8.12, *p*<0.01) and MCT2 (*F*_2,24_=7.43, *p*<0.01) mRNA expression in the hippocampus significantly increased in the Ex group than in the Cont group, whereas these increments were abolished by UK5099 administration before exercise (Figure 4A). After lactate administration, only MCT1 (*F*_2,23_=4.59, *p*<0.05) increased significantly, and MCT2 (*F*_2,23_=2.94, *p*=0.075) tended to be higher than that in the Cont group; however, the increment of those genes was not observed in mice injected UK5099 compared with the Cont group (Figure 4B). On the other hand, no changes were observed in MCT4 mRNA expression after exercise and lactate administration (Figures 4A,B).

### The Effects of Glycogenolysis Inhibition During Exercise on Hippocampal Lactate and the Expression of PGC-1α and MCTs mRNA

Mice received a micro-injection of DAB or vehicle into the hippocampus, and 15min later, mice were subjected to a single bout of high-intensity exercise (Figure 5A). Mice injected with vehicle or DAB showed a similar running distance to fatigue (Figure 4B). Immediately after exercise, the blood lactate levels were similar with and without DAB injection (*F*_2,17_=19.02, *p*<0.0001; Figure 5C). The hippocampal glycogen levels significantly decreased in the Ex group compared with the Cont mice. Still, significant alterations were not noted in the DAB + Ex group, indicating the pre-exercise micro-injection of DAB inhibited hippocampal glycogenolysis during exercise (*F*_2,15_=6.03, *p*<0.05; Figure 5D). The glycogen concentration in mice soleus muscles from the Ex and DAB + Ex groups were decreased compared with Cont group (*F*_2,15_=48.01, *p*<0.0001; Figure 5E). However, liver glycogen concentration did not significantly decrease in either group during a single short-term, high-intensity exercise for approximately 30min (Figure 5F).

Unexpectedly, despite the inhibition of hippocampal glycogenolysis during exercise by DAB, hippocampal lactate levels immediately after exercise showed a similar significant increase in both Ex and DAB + Ex groups. These results indicated that the increase in hippocampal lactate during high-intensity exercise could much depend on lactate uptake from the circulating blood than intra-hippocampal lactate production by astrocyte glycolysis (*F*_2,15_=6.57, *p*<0.01; Figure 5G). Additionally, in the soleus muscle, lactate levels noted immediately after a single bout of high-intensity exercise were significantly higher in both exercise groups than in the Cont group (*F*_2,15_=8.52, *p*<0.01; Figure 5H). Furthermore, the exercise groups with or without DAB injection showed a similar higher PGC-1α (*F*_2,17_=5.29, *p*<0.05), MCT1 (*F*_2,17_=5.58, *p*<0.05), and MCT2 mRNA expression (*F*_2,17_=5.88, *p*<0.05) in the hippocampus than the Cont group, but MCT4 mRNA expression did not differ among the three groups (Figure 5I). These results suggested that the single short-term, high-intensity exercise used in this study rapidly increases lactate release from skeletal muscle into blood, which is pivotal to increase hippocampal lactate concentration and might contribute to enhance the induction of PGC-1α and MCTs mRNA expression in the hippocampus.

## Discussion

Regular exercise training enhances skeletal muscle and hippocampal PGC-1α mRNA and mtDNA copy number (Zhang et al., 2012; Halling et al., 2019). Six weeks of HIIT accompanied by increased blood and hippocampal lactate concentrations has been reported to increase hippocampal expression of mitochondrial biogenesis-related genes PGC-1α, NRF2, and mtDNA copy number (Hu et al., 2021). The effects of regular exercise reflect an accumulation of physiological adaptations produced by bouts of high-intensity exercise. Thus, it is essential to understand the beneficial effects of a single bout of high-intensity exercise and elucidate the mechanisms underlying their effects to produce better high-intensity exercise training strategies. However, the effects of acute high-intensity exercise on hippocampal mitochondrial biogenesis are unclear.

In the current study, an increase in hippocampal PGC-1α mRNA expression was observed 12h after exercise. An increase in hippocampal mtDNA copy number followed 48h later, and its increment depended on exercise intensity. Running time to exhaustion (26.1±0.37min) is slightly below in the high-intensity exercise group compared to the low- and moderate-intensity exercise groups (30min); running distance in the high-intensity group did not differ from that of the moderate-intensity exercise group. However, the blood lactate concentrations immediately after exercise and post-exercise hippocampal mitochondrial biogenesis significantly differed between the three exercise groups. These results are consistent with the previous reports that 20min of a single bout of high-intensity exercise induced the activation of skeletal muscle mitochondrial transcription (Hoshino et al., 2015), and exercise intensity affects skeletal muscle PGC-1α protein levels (Terada et al., 2005; Tadaishi et al., 2011). Moreover, the present results are consistent with the results of the human study, which indicated that a high-intensity interval cycle ergometer for a total duration of 20min showed higher BDNF serum concentration levels than at rest and moderate-intensity exercise group in the human study (Saucedo Marquez et al., 2015).

A blood lactate concentration >12mM was required to increase hippocampal PGC-1α mRNA and mtDNA copy number. In contrast, low- or moderate-intensity exercise correlated with 3–4mM blood lactate concentration, which did not increase the hippocampal PGC-1α mRNA and mtDNA copy number. These results are consistent with previous findings that exercise intensity above the LT increased PGC-1α mRNA expression in human skeletal muscle but that below the LT did not (Tobina et al., 2011). Thus, these results suggested that elevated blood lactate induced by high-intensity exercise above the LT would contribute to the induction of mtDNA copy number through PGC-1α expression in the hippocampus.

To our knowledge, the current study is the first to show that an increase in mitochondrial biogenesis in the brain, especially the hippocampus, depends on exercise intensity in the same manner as seen in skeletal muscle. In contrast, a single bout of low- or moderate-intensity exercise did not affect mitochondrial biogenesis in the hippocampus. Thus, single bouts of low- or moderate-intensity exercise are not likely to induce hippocampal mitochondrial biogenesis, suggesting that these exercises would need to be regularly repeated over 4weeks to promote hippocampal mitochondrial biogenesis (Steiner et al., 2011; Zhang et al., 2012).

Brain cell MCTs transport lactate across the BBB (El Hayek et al., 2019), which is enhanced during exercise (Quistorff et al., 2008; van Hall et al., 2009). Acute (Takimoto and Hamada, 2014) and chronic exercise (El Hayek et al., 2019) above LT enhances MCT expression in the hippocampus, enhancing cognitive function by increasing BDNF expression through the induction of PGC-1α (El Hayek et al., 2019). Besides, Overgaard et al. (2012) indicated that the brain uptakes lactate from the blood and releases lactate into the blood concomitantly, and that net lactate uptake is a plus, and net brain lactate uptake is increased by the increase in circulating blood lactate level during high-intensity exercise. Based on these data, we predicted that some of the blood lactate increased by a single bout of high-intensity exercise or lactate administration would be taken up into the brain *via* MCTs, where it would promote PGC-1α expression, followed by mitochondrial biogenesis. To test this hypothesis, we examined hippocampal extracellular lactate concentration by using *in vivo* microdialysis in the presence and absence of UK5099, a potent MCT1–4 inhibitor, after lactate injection. In this experiment, to eliminate the influence of lactate production derived from astrocytes glycogenolysis, which would be expected to occur during high-intensity exercise, I.P. lactate administration and microdialysis were performed using the resting instead of the exercised mice.

We found that the hippocampal extracellular lactate concentration increased following lactate injection, consistent with previous studies (Machler et al., 2016; Carrard et al., 2018). However, this lactate concentration increase was suppressed by UK5099 administration, indicating that MCT inhibition prevents lactate transport from the blood into hippocampal extracellular space. Furthermore, UK5099 administration also suppressed lactate-induced increases in hippocampal PGC-1α and TFAM mRNA, and mtDNA copy number.

The results demonstrate that high-intensity exercise or lactate administration increases hippocampal extracellular lactate concentration, which could induce hippocampal PGC-1α mRNA expression and increase mtDNA copy number. Moreover, hippocampal BDNF mRNA expression was also increased after a single bout of high-intensity exercise, which was consistent with a previous study showing that increased PGC-1α led to the induction of BDNF expression (Wrann et al., 2013; Mikami et al., 2021). In addition, the lactate-induced hippocampal BDNF expression was suppressed by injecting MCT1/2 inhibitor, AR-C155858 (El Hayek et al., 2019). However, the present study does not elucidate how the increased hippocampal lactate upregulated PGC-1α and BDNF mRNA expression. Yang et al. showed that exposure of neurons to lactate modified the intracellular NADH/NAD ratio, promoted N-methyl-D-aspartate (NMDA) receptor activity and its downstream signaling cascade Erk1/2 (Yang et al., 2014). The activating of the NMDA receptors caused an increase in intracellular Ca^2+^ concentration, thereby inducing the gene expression of PGC-1α (Luo et al., 2009). We hypothesize that lactate taken up from blood into the brain could have altered intracellular NADH/NAD ratio and could have triggered changed mRNA expressions related to mitochondrial biogenesis. Further studies are necessary to prove our hypothesis.

Exogenous lactate administration increased blood lactate concentration, induced hippocampal PGC-1α and MCT mRNA expression, and increased mtDNA copy number, similar to high-intensity exercise. These results indicated that systemic administration of lactate mimics the effect of high-intensity exercise on hippocampal mitochondria biogenesis. However, a single lactate administration was insufficient to increase MCT2 and BDNF mRNA, and mtDNA copy number to the same extent as a single bout of high-intensity exercise.

We speculate that the differences in hippocampal mRNA expression and mtDNA copy number between exogenous lactate administration and bouts of high-intensity exercise may result from the production of myokines and other factors induced by high-intensity exercise but not by exogenous lactate administration. Irisin, a myokine released from skeletal muscle, is transferred to the brain and induces hippocampal PGC-1α and BDNF expression (Wrann et al., 2013; Azimi et al., 2018; Lourenco et al., 2019). In the future studies, we plan to investigate the effect of lactate administration on myokines in the circulating blood.

The role of lactate produced by astrocytic glycolysis in mediating memory processes (Descalzi et al., 2019) and exercise endurance capacity has recently been characterized (Machler et al., 2016; Matsui et al., 2017). The glycogen in astrocytes is broken down and metabolized to lactate, shuttled to neurons *via* MCT2, and acts as an energy source in nerve cells (Pellerin et al., 1998; Brooks, 2020). Inhibiting hippocampal glycogenolysis *via* DAB injection impaired memory and decreased endurance exercise capacity, indicating that the lactate supplied by astrocytes to neurons is critical for regulating memory processing (Suzuki et al., 2011) and maintaining endurance capacity (Matsui et al., 2017). We speculate that glycogen-derived lactate released during exercise might mediate the promotion of hippocampal mitochondrial biogenesis after high-intensity exercise.

Our results showed that compared with mice injected with saline, DAB injection before exercise inhibited hippocampal glycogenolysis after exercise; however, hippocampal lactate concentration following high-intensity exercise was elevated in the presence or absence of DAB. Moreover, hippocampal PGC-1α, MCT1, and MCT2 mRNA expression following high-intensity exercise were unaffected by DAB injection before exercise. These results indicate that the lactate derived from hippocampal glycogenolysis during high-intensity exercise does not promote hippocampal PGC-1α, MCT1, and MCT2 mRNA expression. This was inconsistent with the previous findings that lactate derived from astrocytic glycogenolysis is vital for cognitive memory and regulating exercise performance. Based on these results, although a possible effect of brain glycogen-derived lactate release cannot be ruled out, we speculate that the induction of hippocampal PGC-1α, MCT1, and MCT2 mRNA expression after exhaustive and short-term exercise is attributable to the marked increase in the lactate taken into the brain from the blood, rather than from astrocytic glycogenolysis.

The following results support our speculation; (1) hippocampal extracellular lactate, taken up from blood into the brain, was suppressed by MCT inhibitors, followed by a marked decrease in PGC-1α, MCT1, and MCT2 mRNA expression; (2) the inhibition of hippocampal glycogenolysis by DAB did not affect PGC-1α, MCT1, and MCT2 mRNA expression. The type of exercise used in the present study was short-term and exhaustive; if the exercise were different, the contribution of lactate derived from brain glycogenolysis on gene expression related to mitochondrial biogenesis would be altered. Further study is necessary to investigate the contribution of lactate, which is incorporated from circulating blood or produced from brain glycogenolysis, and the beneficial effect of different types of exercises on brain function. Our results provide a helpful strategy to enhance brain function *via* high-intensity exercise training, which may contribute to developing exercise programs in human subjects.

### Limitations

In the present study, we used only male mice, based on results from a previous study using male mice that showed that intermittent intense exercise prevents the stress-induced decline of cognitive function and suppresses the decrease in neurons survival (Lee et al., 2018). Thus, we did not compare the difference in hippocampal mitochondrial biogenesis after exercise between male and female mice. Therefore, the sex difference in mitochondrial biogenesis is not clarified. However, it has been reported that there are no differences in heart mitochondrial activity between sexes in young mice (6weeks old). On the other hand, it has been reported that female mice have a higher brain mitochondrial respiratory and reserve function than male mice because of lower H_2_O_2_ production in female cardiac and brain tissue (Khalifa et al., 2017). Besides, it has been reported that, in old age mice (22months old), the gene expression of key regulators of mitochondrial biogenesis (PGC-1α, Sirtuin1, and NRF2) and mitochondrial activity in the brain was significantly lower in females than males (Zawada et al., 2015). Until now, the sex differences in the effects of exercise on brain mitochondrial biogenesis have not been clarified. Therefore, further studies to examine whether mitochondrial biogenesis induced by high-intensity exercise is different between the sexes are needed.

This study focused only on lactate and did not consider stress-related factors, such as cortisol, (nor) epinephrine, and corticosterone. High-intensity exercise, such as the one used in the present study, increases the stress-related factors mentioned above. Chronic stress has previously been shown to have undesirable effects on brain functions. On the other hand, some studies have reported that acute stress, unlike chronic stress, has a beneficial effect on brain functions. For example, acute and short-term stress or single doses of corticosterone significantly increase hippocampal cell proliferation (Kirby et al., 2013). Additionally, acute stress increases the circulating corticosterone levels, leading to the activation of glucocorticoid receptors and subsequently provoking the synthesis and BDNF expression (Yang et al., 2004). Based on these results, stress-related factors might have also contributed to the increased mitochondrial biogenesis observed in this study. However, we did not examine the effects of high-intensity exercise-induced mitochondria biogenesis on stress-related factors such a corticosterone plasma concentration and PGC-1α alteration in the presence of corticosterone antagonist. Therefore, in addition to the effect of blood lactate on mitochondria biogenesis, the study of stress-related factors on increased mitochondria biogenesis after high-intensity exercise is necessary for the future.

## Conclusion

Our results show that a single bout of exercise above the LT intensity enhanced hippocampal mitochondrial biogenesis. A single injection of exogenous lactate increased hippocampal extracellular lactate concentration, which partially mimicked the effects of a single bout of high-intensity exercise. Additionally, the UK5099 administration abolished the increase in hippocampal extracellular lactate concentration. However, DAB did not affect hippocampal lactate, PGC-1α, and MCT mRNA concentration despite inhibiting hippocampal glycogenolysis. Therefore, the lactate released into the blood from skeletal muscle during high-intensity exercise may be a crucial stimulator for hippocampal mitochondria biogenesis. These data improve our understanding of the effects of high-intensity exercise and may be a basis for further clinical or athletic applications.

## Data Availability Statement

The raw data supporting the conclusions of this article will be made available by the authors, without undue reservation.

## Ethics Statement

The animal study was reviewed and approved by the Animal Care and Use Committee of Nippon Medical School (approval no. 30-030).

## Author Contributions

JP and TM conceived and designed experiments, interpreted the results of experiments, and drafted the manuscript. JP, JK, and TM performed experiments and analyzed data. JP prepared figures. All authors have approved to submit the final version manuscript.

## Funding

This study was supported by a Grant-in-Aid for Early-Career Scientists (19K20051) from the Japan Society for the Promotion of Science (JSPS).

## Conflict of Interest

The authors declare that the research was conducted in the absence of any commercial or financial relationships that could be construed as a potential conflict of interest.

## Publisher’s Note

All claims expressed in this article are solely those of the authors and do not necessarily represent those of their affiliated organizations, or those of the publisher, the editors and the reviewers. Any product that may be evaluated in this article, or claim that may be made by its manufacturer, is not guaranteed or endorsed by the publisher.
